# Temperature and Resources Interact to Affect Transmission via Host Foraging Rate and Susceptibility

**DOI:** 10.1111/ele.70151

**Published:** 2025-06-24

**Authors:** Daniel C. Suh, Katie Schroeder, Alexander T. Strauss

**Affiliations:** ^1^ Odum School of Ecology University of Georgia Athens Georgia USA; ^2^ Center for the Ecology of Infectious Diseases University of Georgia Athens Georgia USA

**Keywords:** *Daphnia*, disease ecology, exposure, foraging, modelling, resources, susceptibility, temperature, transmission

## Abstract

Environmental conditions such as temperature and resource availability can shape disease transmission by altering contact rates and/or the probability of infection given contact. However, interactive effects of these factors on transmission processes remain poorly understood. We develop mechanistic models and fit them to experimental data to uncover how temperature and resources jointly affect transmission of fungal parasites ( *Metschnikowia bicuspidata* ) in zooplankton hosts (*Daphnia dentifera*). Model competition revealed interactive effects of temperature and resources on both contact rates (host foraging) and the probability of infection given contact (per‐parasite susceptibility). Foraging rates increased with temperature and decreased with resources (via type‐II functional response), but this resource effect weakened at warmer temperatures due to shorter handling times. Per‐parasite susceptibility increased with resources at cooler temperatures but remained consistently high when warmer. Our analysis demonstrates that temperature and resources interact to shape transmission processes and provides a general theoretical framework for other host–parasite systems.

## Graphical abstract

Environmental conditions such as temperature and resource availability can shape disease transmission by altering contact rates and/or the probability of infection given contact. We develop mechanistic models and fit them to experimental data to uncover how temperature and resources jointly affect transmission of fungal parasites ( *Metschnikowia bicuspidata* ) in zooplankton hosts (Daphnia dentifera). Our analysis demonstrates that temperature and resources interact to shape transmission processes and provides a general theoretical framework for other host–parasite systems.

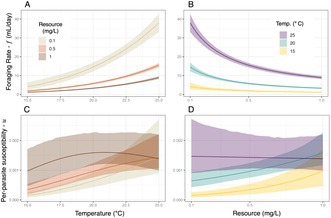

## 1 Introduction

Transmission underlies all infectious diseases, and how it responds to environmental conditions remains a primary objective of infectious disease research (King et al. 2023; Rohr et al. 2011). Transmission can be broken down into two processes: contact between a susceptible host and parasite (either in the environment or in an infected host) and the probability of infection given contact (i.e., per‐parasite susceptibility for environmental transmission). Traditionally, these two processes have been modelled as a single transmission parameter beta, *β* (McCallum 2001), but disentangling them permits more mechanistic understanding of transmission (Civitello and Rohr 2014; Kirk et al. 2019; McCallum et al. 2017; Strauss et al. 2019). Here, we investigate how each process responds separately to environmental change.

In an era of enhanced anthropogenic disturbances, there is substantial interest in understanding how disease transmission responds to changes in both temperature (Altizer et al. 2013; Baker et al. 2022; Lafferty 2009; Mordecai et al. 2017) and resource availability (Becker et al. 2015; Gottdenker et al. 2014). Each factor is well known to affect contact rates (Hall et al. 2007; Kirk et al. 2020; Shocket et al. 2018), the probability of infection given contact (Hawley and Altizer 2011; Strauss et al. 2021), and overall disease transmission (Becker et al. 2015; Hurtado et al. 2014; Mordecai et al. 2017; Rohr and Cohen 2020). However, joint effects of temperature and resources have received less attention, and it is unclear how these factors might interact.

Temperature affects the epidemiology of a wide range of host species, including monarch butterflies (Ragonese et al. 2024), amphibians (Paull and Johnson 2014), and corals (Burke et al. 2023). Changes in temperature can alter transmission via contact rates (Shocket et al. 2018) or the probability of infection given contact (Kirk et al. 2020; Shocket et al. 2019). Warmer temperatures can elevate contact rates by increasing the aggregation of hosts (Martinez et al. 2024) or biting rates of vectors (Mordecai et al. 2017). For ectothermic hosts that encounter parasites while foraging, temperature can increase contact rates by accelerating host foraging (Hopkins et al. 2018; Shocket et al. 2018; Thongsripong et al. 2021). Temperature can also alter the probability of infection given contact by either reducing (Brand et al. 2016) or enhancing host immunity (Catalán et al. 2012) or altering the evolution of host defence (Dziuba et al. 2023). The context‐dependent nature of temperature effects on transmission emphasises the need to characterise these effects across a range of other environmental contexts.

Resource availability also affects both contact rates and the probability of infection given contact. For example, anthropogenic resource provisioning can cause host aggregations that elevate contact rates between susceptible and infected hosts (Becker and Hall 2014). When hosts encounter parasites in the environment while foraging, higher resources can reduce exposure (Hall et al. 2007) because foraging rate (defined as the volume or area of habitat searched per unit time) declines with resources in type‐II and type‐III functional responses (Holling 1965). Hosts may also invest more into immune defence—reducing the probability of infection given contact—when resources are more plentiful (Budischak et al. 2018; Houston et al. 2007; Strandin et al. 2018). Thus, resources (and temperature) can each affect transmission by altering contact rates and/or the probability of infection given contact.

Despite strong foundations of independent effects of temperature and resources on disease, their joint effects on transmission are understudied. Previous experiments and theory have integrated effects of temperature into transmission models based on foraging (Shocket et al. 2018, 2019), but the additive or interactive effects of temperature and resources have not been explored. Anthropogenic forces simultaneously affect resources and temperature, emphasising the importance of studying the joint effects of these concurrent global changes on disease (Bradley and Altizer 2007; Martin et al. 2010). Other fields, such as consumer‐resource dynamics, have begun to explore consequences of these interactions (Huey and Kingsolver 2019; Thomas et al. 2017), but their findings have not yet been extended to disease transmission.

Here, we develop and test models to explore how temperature and resources interact to affect the transmission of fungal parasites ( *Metschnikowia bicuspidata* ) in a freshwater zooplankton host (*Daphnia dentifera*). *Daphnia* are filter‐feeders that indiscriminately consume algal resources and fungal spores while foraging (Hall et al. 2007). We tested how host foraging rates and infection prevalence varied over field‐relevant temperature and resource gradients and then developed models to articulate how these factors might affect transmission. We hypothesised that contact rates would increase with temperature and decrease with resources, since these ectothermic hosts exhibit type‐II or III functional responses (McCauley et al. 1990; Sarnelle and Wilson 2008). We hypothesised that the probability of infection given contact would increase with temperature due to increased infectivity of the parasite (Auld and Brand 2017; Shocket et al. 2018) but decrease with higher resources via enhanced host immunity. Finally, we hypothesised that temperature and resources might interact if, for example, host functional responses or resource‐dependent immune function varied with temperature. Model competition revealed that temperature and resources interacted to shape both contact rates (i.e., foraging rate) and the probability of infection given contact (i.e., per‐parasite susceptibility). Foraging rate increased with temperature and decreased with resources as predicted, and an interaction emerged due to temperature‐dependent handling times. Per‐parasite susceptibility increased with temperature, but only at low and medium resource levels. This model‐experiment combination clarifies how temperature and resources interact to regulate transmission in this model system and provides a mathematical template on which to build parallel models tailored to other disease systems.

## 2 Methods

### 2.1 Study System

The zooplankton host *Daphnia dentifera* and fungal parasite *Metschnikowia bicuspidata* are a highly tractable system for studying how biotic and abiotic factors influence transmission (Ben‐Ami et al. 2008; Hall et al. 2007; Strauss et al. 2019). *D. dentifera* are non‐selective grazers and can become infected when they inadvertently consume parasite spores in the water. Daphniid hosts have immune protection in the form of a physical barrier (the gut wall epithelium) and the presence of host haemocytes that can degrade fungal spores that penetrate the gut (Stewart Merrill et al. 2019; Sun et al. 2023). Consequently, transmission is the product of foraging rate (i.e., contact rate) and the susceptibility of the host per spore encountered (i.e., per‐parasite susceptibility). Hosts rarely clear infections after the fungus has begun to reproduce within the host, and host individuals do not shed fungal spores until host death.

### 2.2 Laboratory Conditions and Experimental Setup

All hosts were maintained in a mixture of 10% filtered lake water (1 μm Pall A/E) and 90% treated tap water (passed through activated carbon) in constant‐temperature incubators (22°C) with a standardised light cycle (16 h light; 8 h dark). *D. dentifera* reproduce parthenogenetically, and all individuals used in these experiments originated from a single wild‐caught and subsequently lab‐reared isoclonal line from MI, USA (Hall et al. 2007). We fed hosts laboratory‐maintained stocks of a high‐quality green algae, *Ankistrodesmus falcatus* (Supplementary Methods S1). Hosts were fed at 1.0 mg dry mass algae per liter of water (mg/L) for at least three generations prior to the experiments to standardise any potential maternal effects. Parasite spores were reared in vivo in the same isoclonal host line and used within 2 weeks for the infection assay.

### 2.3 Foraging Rate Assay

We measured the foraging rate (i.e., contact rate) of hosts (*n* = 30) under nine different treatment conditions: three temperatures (15°C, 20°C, 25°C) crossed with three resource concentrations (0.1, 0.5, 1.0 mg/L). These treatments represent realistic ranges encountered in nature and build upon previous experimental work in this system (Hall et al. 2009; Manzi et al. 2020; Shocket et al. 2018, 2019). Hosts were harvested as neonates within a 24‐h window and housed individually in 15 mL conical vials (tubes). Their treatment conditions were established within 24 h, creating variation in body size by the time the foraging assay began (Day 5). This variation is important because foraging rate is proportional to the surface area (i.e., body length‐squared) of the host (Figure S1) (Hall et al. 2007; Strauss et al. 2019). At 5 days, we estimated foraging rates by comparing resource concentrations between treatment tubes (grazed; algae and *Daphnia*) and control tubes (ungrazed; only algae) after an 8‐h foraging period via relative measurements of in vivo fluorescence. The entire assay was conducted in the dark to prevent algae growth, so that all differences in fluorescence arose from foraging alone, and tubes were inverted every half hour to resuspend algae. At the end of the assay, we removed hosts and measured their length. If hosts were unable to be measured, then we interpolated length as the mean body size from that treatment (Supplementary Methods S2). In vivo fluorescence values were converted back into units mg/L via linear regression (Supplementary Methods S1), resulting in an estimate of the quantity of resources consumed during the assay.

### 2.4 Infection Assay

The infection assay was conducted over 6 weeks with identical treatments to the foraging rate assay. We harvested hosts as neonates within a 24‐h window and maintained them at their respective treatment conditions for 5 days. At day 5 of the experiment, we isolated individual hosts into separate 50 mL conical vials (tubes) and exposed them to *M. bicuspidata* fungal spores at a concentration of 200 spores/mL for 24 h. We exposed 30 individuals per treatment at 20°C and 25°C and 45 individuals per treatment at 15°C. Tubes were inverted every 6 h to resuspend spores. A separate group of hosts (*n* = 7–10) from each treatment was measured at Day 5 to estimate mean body length at the time of exposure (Figure S1). Since extra hosts were not available for the 0.5 mg/L treatment at 25°C, mean length was interpolated as the average between the 0.1 and 1.0 mg/L treatments at 25°C (Methods S2). Each day of the infection assay, we refreshed the water and resources in each tube, removed any neonates, and checked for host mortality. Upon death, we checked whether hosts were infected, providing us with data on infection prevalence across all treatments. We removed hosts that died before infections could be reliably detected, which resulted in uneven sample sizes across treatments (*n* = 17–40; Table S1).

### 2.5 Traditional Statistics

All analyses were conducted in R (version 4.3.1). We used traditional statistics to test whether three response variables (body length and resources consumed from the foraging assay; infection prevalence from the infection assay) varied with continuous predictors: temperature, resource concentration, or their interaction. For body length and resources consumed, we used linear regressions. For infection prevalence, we used a generalised linear model with a logit‐link function. We tested whether the effects of temperature, resources, or their interaction were significant. When interactions were significant, we performed post hoc linear regressions to ask how resources affected the response variable separately at each temperature.

### 2.6 Model Competition and Parameterization

We focus the bulk of our analysis on building a suite of mechanistic models, estimating parameter values to fit these models to empirical data, and competing models against each other using AIC. The models used ordinary differential equations to track the quantity of resources consumed (Model Competition 1) and the conversion of hosts from susceptible to infected (Model Competition 2). The model fitting reveals how the processes of contact (Model Competition 1) and the probability of infection given contact (Model Competition 2) vary with the combination of resources and temperature. The model competition determines the most parsimonious explanation of the observed data. Definitions and units of state variables and parameters are included in the Supporting Information (Table S2).

### 2.7 Model Competition 1: Foraging Rate

We first focus on the process of contact (i.e., host foraging) and begin with a simple foraging model to describe changes in resource quantity over time in the foraging assay:

(1) $$\frac{\mathrm{dR}}{\mathrm{dt}}=-\mathrm{fRN}$$

where R represents the quantity of the resource (algae), N represents the number of hosts, and f is the per‐capita host foraging rate (sometimes called ‘clearance rate’ for filter feeders like *Daphnia*). We developed and compared five mechanistic models to specify different effects of temperature and/or resources on these foraging dynamics by modifying f.

These foraging models include (1A) size‐only; (1B) temperature‐only; (1C) resource‐only; (1D) additive resource and temperature; and (1E) interactive resource and temperature. The simplest model (1A) introduces a power function for host surface area (body length *L*‐squared) that is included in all subsequent models, creating a size‐specific foraging rate $f^{\prime }$ and allowing foraging rate to increase with host size. In model 1B, foraging rate increases exponentially with temperature according to its Arrhenius coefficient $T_{A}^{f}$, relative to a reference temperature $T_{R}$, capturing the increasing portion of a thermal reaction norm. Resource effects are included in model 1C with a type‐II functional response, formulated with handling time h. The ‘additive’ model (1D) combines temperature and resource effects as specified in 1B (numerator of 1D) and 1C (denominator of 1D). The ‘interactive’ model (1E) modifies the additive model by allowing handling time $h^{\prime }$ to vary with temperature according to an exponential slope coefficient ω, thereby making the resource effect dependent on temperature (Supplementary Methods S3).

(1A) $$f=f^{\prime }L^{2}$$

(1B) $$f=f^{\prime }L^{2}e^{T_{A}^{f}\left(1/T_{R}-1/T\right)}$$

(1C) $$f=\frac{f^{\prime }L^{2}}{1+f^{\prime }L^{2}\mathrm{hR}}$$

(1D) $$f=\frac{f^{\prime }L^{2}e^{T_{A}^{f}\left(1/T_{R}-1/T\right)}}{1+f^{\prime }L^{2}e^{T_{A}^{f}\left(1/T_{R}-1/T\right)}\mathrm{hR}}$$

(1E) $$f=\frac{f^{\prime }L^{2}e^{T_{A}^{f}\left(1/T_{R}-1/T\right)}}{1+f^{\prime }L^{2}e^{T_{A}^{f}\left(1/T_{R}-1/T\right)}h^{\prime }e^{\omega T}R}$$

For each model in Competition 1, we simulated Equation (1) for the duration of the foraging assay (8 h) using the ‘deSolve’ (v1.36) package (Soetaert et al. 2008), with starting resource concentrations as in the experiment and body size as measured for each host. We estimated all other model parameters by maximum likelihood using the ‘bbmle’ (v1.0.25) package (Bolker and R Development Core Team 2007). We assumed that square root‐transformed resource concentrations were normally distributed and estimated their standard deviation (*σ*) as part of the model fitting process. Parameter estimates were chosen that maximised the likelihood of observing the data (resources remaining) given each model formulation (Rosenbaum and Rall 2018). Finally, we simulated each model with its fitted parameters over our experimental gradients to plot them graphically. When necessary, we interpolated data via linear regression (e.g., body size, Supplementary Methods S2), or used mean values (e.g., duration of assay).

### 2.8 Model Competition 2: Per‐Parasite Susceptibility

Next, we incorporated the best foraging model from Model Competition 1 (1E; see Results) into a suite of models to incorporate effects of temperature and resources on the probability of infection given contact (i.e., per‐parasite susceptibility). To limit the number of competing models, we restrict our foraging model to the top performer from Model Competition 1; however, we re‐fit all parameters with each model in Model Competition 2. In Competition 2, a system of differential equations describes changes in the densities of resources (*R*), parasite spores (*Z*), and the conversion of susceptible hosts (*S*) into infected hosts (*I*) during the infection assay:

(2) $$\frac{\mathrm{dR}}{\mathrm{dt}}=-\mathrm{fR}\left(S+I\right)$$

$$\frac{\mathrm{dS}}{\mathrm{dt}}=-\text{ufZS}$$

$$\frac{\mathrm{dI}}{\mathrm{dt}}=\text{ufZS}$$

$$\frac{\mathrm{dZ}}{\mathrm{dt}}=-\mathrm{fZ}\left(S+I\right)$$

Each experimental unit included a single host so S and I are fractional and represent the probability of occurring in either state over time. Transmission is a function of exposure (contact rate; f), susceptibility of the host per spore consumed (per‐parasite susceptibility; u), the density of susceptible hosts (S), and the density of spores (Z).

The transmission models (2A–2E) differ in their formulation of per‐parasite susceptibility (u in Equation 2): (2A) independent; (2B) temperature‐only; (2C) resource‐only; (2D) additive resource and temperature; and (2E) interactive resource and temperature (detailed explanation of each model is included in Supplementary Methods S4). As a baseline, model 2A assumes per‐parasite susceptibility is similar across all treatments. Model 2B allows $u^{\prime }$ to vary with temperature according to the Arrhenius coefficient $T_{A}^{u}$. Model 2C allows $u^{\prime }$ to vary exponentially with resource conditions instead. Since we expect per‐parasite susceptibility to depend more on lifetime accumulation of resources by the host than instantaneous resource levels during exposure, we specify that u’ varies with resource treatment levels ([$R_{\mathrm{trt}}]$) rather than dynamically changing resources (R). Model 2D combines the functions from 2B and 2C into additive effects of temperature and resources. Finally, model 2E adds an interaction term ϕ that allows $u^{\prime }$ to scale exponentially by the product of temperature and resources.

(2A) $$u=u^{\prime }$$

(2B) $$u=u^{\prime }e^{T_{A}^{u}\left(1/T_{R}-1/T\right)}$$

(2C) $$u=u^{\prime }e^{\rho \left[R_{\mathrm{trt}}\right]}$$

(2D) $$u=u^{\prime }e^{T_{A}^{u}\left(1/T_{R}-1/T\right)+\rho \left[R_{\mathrm{trt}}\right]}$$

(2E) $$u=u^{\prime }e^{T_{A}^{u}\left(1/T_{R}-1/T\right)+\rho \left[R_{\mathrm{trt}}\right]+\phi \left[R_{\mathrm{trt}}\right]T}$$

We parameterised each transmission model with data from both the infection assay and foraging assay via maximum likelihood. Specifically, we simulated the system of equations (Equation 2) with conditions that mimicked the experimental design and selected parameters that maximised the similarity between model outcomes and empirical results (quantity of resources consumed and number of exposed individuals that became infected). For likelihood calculations, we assumed infection outcomes were binomially distributed. Finally, we chose the most parsimonious model according to corrected AIC, incorporating data from both the foraging assay and the infection assay. We calculated confidence intervals around parameter estimates of the best model in Competition 2 via bootstrapping (Table S3; 1005 iterations). A complementary model explored how per‐parasite susceptibility varied with body size (Supplementary Methods S5, Figure S3).

## 3 Results

### 3.1 Traditional Statistics

**FIGURE 1:**
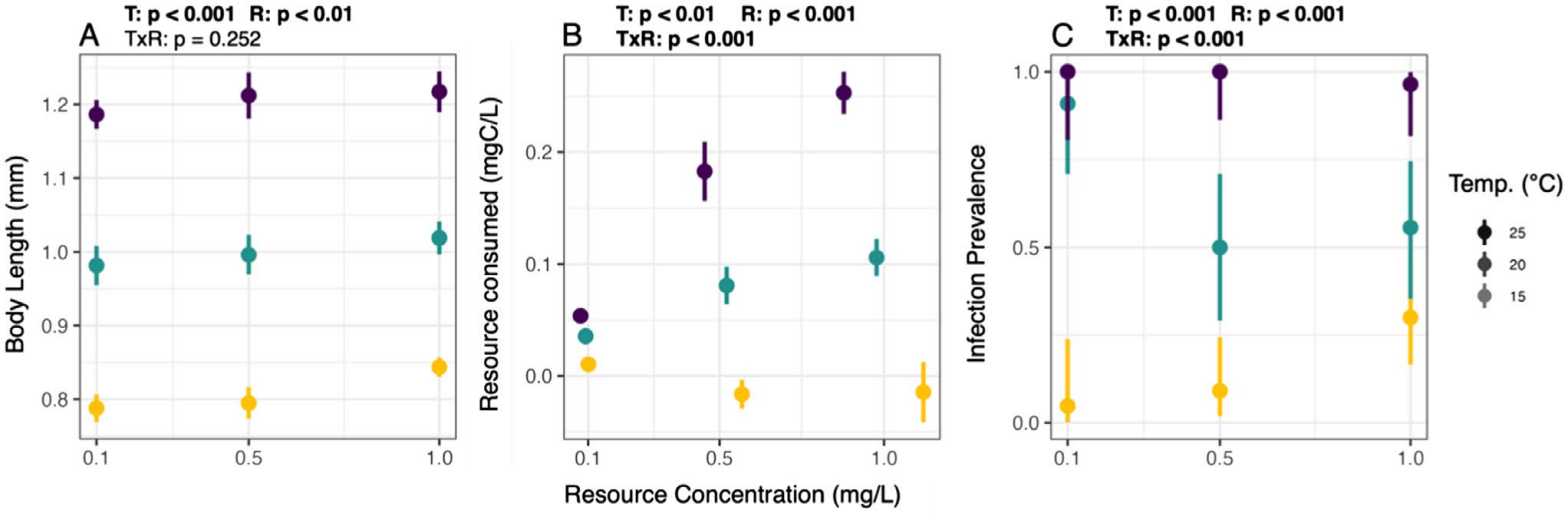
Joint effects of temperature and resources on host body size, resources consumed, and infection prevalence. T, R and TxR stand for temperature, resources, and the interaction between temperature and resources, respectively. These values represent significance results (*p*‐values) from linear models for each variable testing for effect of temperature, resources, and their interaction on the corresponding response variables (body length, resources consumed, or infection prevalence). *p*‐values < 0.05 are bolded. (A) Body length (in millimetres) increased starkly with temperature and weakly with resource concentration. (B) Resources consumed (mg/L) over 8 h during the foraging assay increased with resources at 25°C and 20°C but not 15°C. Limited sensitivity of the fluorometer resulted in mean foraging rates that were not significantly different from 0°C at 15°C. Positions on the *x*‐axis are not aligned perfectly with intended treatment levels because they reflect actual fluorescence readings from the experiment (see Supporting Information S1 for more details). (C) Infection prevalence (number infected/number exposed) was uniformly high at 25°C, decreased with resources at 20°C, and increased with resources at 15°C. All error bars show 95% confidence intervals. Colours represent the different temperature treatments.

Traditional statistics revealed clear effects of both temperature and resource concentration on body length, resources consumed, and infection prevalence (Figure 1, Table S4). Body length of hosts increased with both resource concentration (*p* < 0.01) and temperature (*p* < 0.001), but these factors did not interact (*p* = 0.252). Resources consumed over the 8‐h foraging assay also increased with both resources (*p* < 0.001) and temperature (*p* < 0.01). These factors did significantly interact (*p* < 0.001); post hoc linear regressions at each temperature showed that resources consumed increased steeply with resources at 25°C (slope = 0.218; *p* < 0.001), less steeply at 20°C (slope = 0.007; *p* < 0.001), and did not vary significantly with resources at 15°C (*p* = 0.074). At 15°C, resources consumed were sometimes close to zero or slightly negative because of the limited sensitivity of the fluorometer (Figure 1, middle panel; Supplementary Methods S1). Infection prevalence was highest at 25°C and showed opposing responses to resources in the 20°C and 15°C treatments (Figure 1; rightmost panel). Statistically, these effects of resources and temperature were both positive (*p* < 0.01), with a negative interaction effect (*p* < 0.01). Post hoc linear regressions at each temperature showed that the relationship between infection prevalence and resources was positive at 15°C (slope = 2.62; *p* < 0.01), negative at 20°C (slope = −1.61; *p* < 0.05), and absent at 25°C (*p* = 0.998).

### 3.2 Model Competition 1: Foraging Rate

The interactive foraging rate model (model 1E) was the best performing foraging model and had an AICc weight of 0.9914, despite being the most complex with 5 parameters (Table 1A). Following model 1E, the additive model (model 1D) performed second best with an AICc weight of 0.0086, and all other models performed extremely poorly with AICc weights below 0.001.

**TABLE 1:** (A) Model competition 1 results (foraging rate); (B) Model competition 2 results (per‐parasite susceptibility).

Model	log‐likelihood	AICc	Δ log‐likelihood	ΔAICc	# Parameters	Weight
(A) Competition 1: Foraging rate						
(1E) Interactive	1038.0	−2065.8	125.9	0.0	5	0.9914
(1D) Additive	1032.2	−2056.3	120.1	9.5	4	0.0086
(1B) Temperature‐only	965.8	−1925.5	53.7	140.3	3	< 0.001
(1C) Resource‐only	926.7	−1847.4	14.7	218.4	3	< 0.001
(1A) Size‐only	912.1	−1820.1	0.0	245.6	2	< 0.001
(B) Competition 2: Per‐parasite susceptibility						
(2E) Interactive	947.2	−1876.1	5.8	0.0	9	0.7391
(2D) Additive	944.1	−1872.0	2.7	4.1	8	0.0951
(2C) Resource‐only	942.9	−1871.6	1.5	4.5	7	0.0779
(2A) Independent	941.4	−1870.6	0.0	5.5	6	0.0472
(2B) Temperature‐only	942.2	−1870.3	0.8	5.8	7	0.0407

**FIGURE 2:**
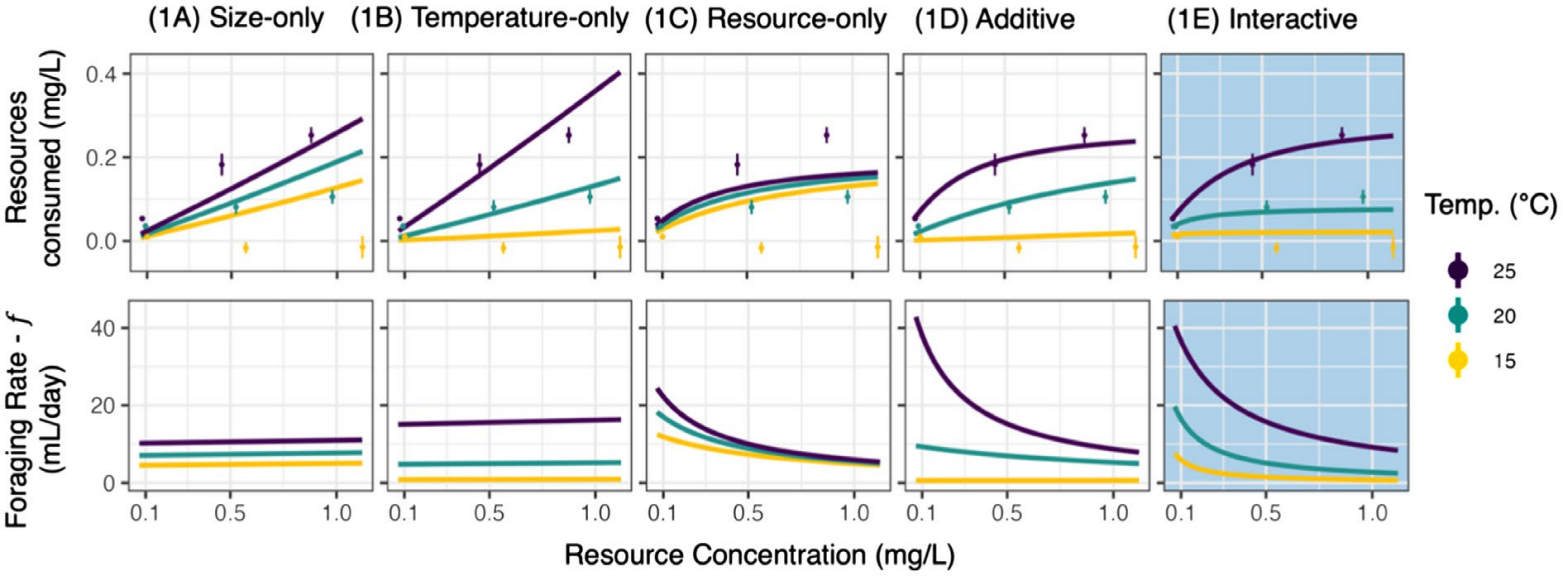
Five separate models from Competition 1 fit to data from the foraging rate assay. Columns show the best fit for each model: (1A) size‐only; (1B) temperature‐only; (1C) resource‐only; (1D) additive; (1E) and interactive. Top Row: Predicted and observed resources consumed in the foraging assay. Bottom row: Model predictions for foraging rate, which determines spores consumed in Model Competition 2 (see Figure 3). Foraging rate is the volume of water filtered by an individual per unit time and is analogous to the contact rate between an individual and fungal spores. The best performing model (1E) is shaded in blue. Error bars for resources consumed depict 95% confidence intervals (+/− 1.96 SE).

The poorest performing model was the size‐only model (model 1A, Figure 2), which was only able to delineate between the treatments based on the size differences among hosts. The resource‐only (model 1C) and temperature‐only (model 1B) models performed slightly better but were unable to respond to both resources and temperature. The additive model performed better than the previous three models (models 1A–1C) and captured both the increases in foraging with temperature and the saturating response across the resource gradient. Finally, the interactive model (model 1E) performed dramatically better than all other models (Figure 2; shaded in blue). The interactive model allowed handling time to decrease exponentially with temperature, magnifying the effect of resources at higher temperatures. Thus, hosts ‘handle’ their resources faster when it is warmer. Taken together, foraging rate—hence contact with parasites—was clearly influenced by both temperature and resources. Moreover, the interaction between temperature and resources demonstrates that these patterns are highly context dependent.

### 3.3 Model Competition 2: Per‐Parasite Susceptibility

The interactive model (model 2E) was the best performing model in Competition 2 with an AICc weight of 0.7391 (Table 1B). The interactive model was the most complex model and had 9 parameters compared to only 6 parameters for the simplest transmission model. In addition to including interactive effects of temperature and resources on host foraging (included in models 2A–2E), it also included interactive effects of temperature and resources on per‐parasite susceptibility (only included in 2E).

**FIGURE 3:**
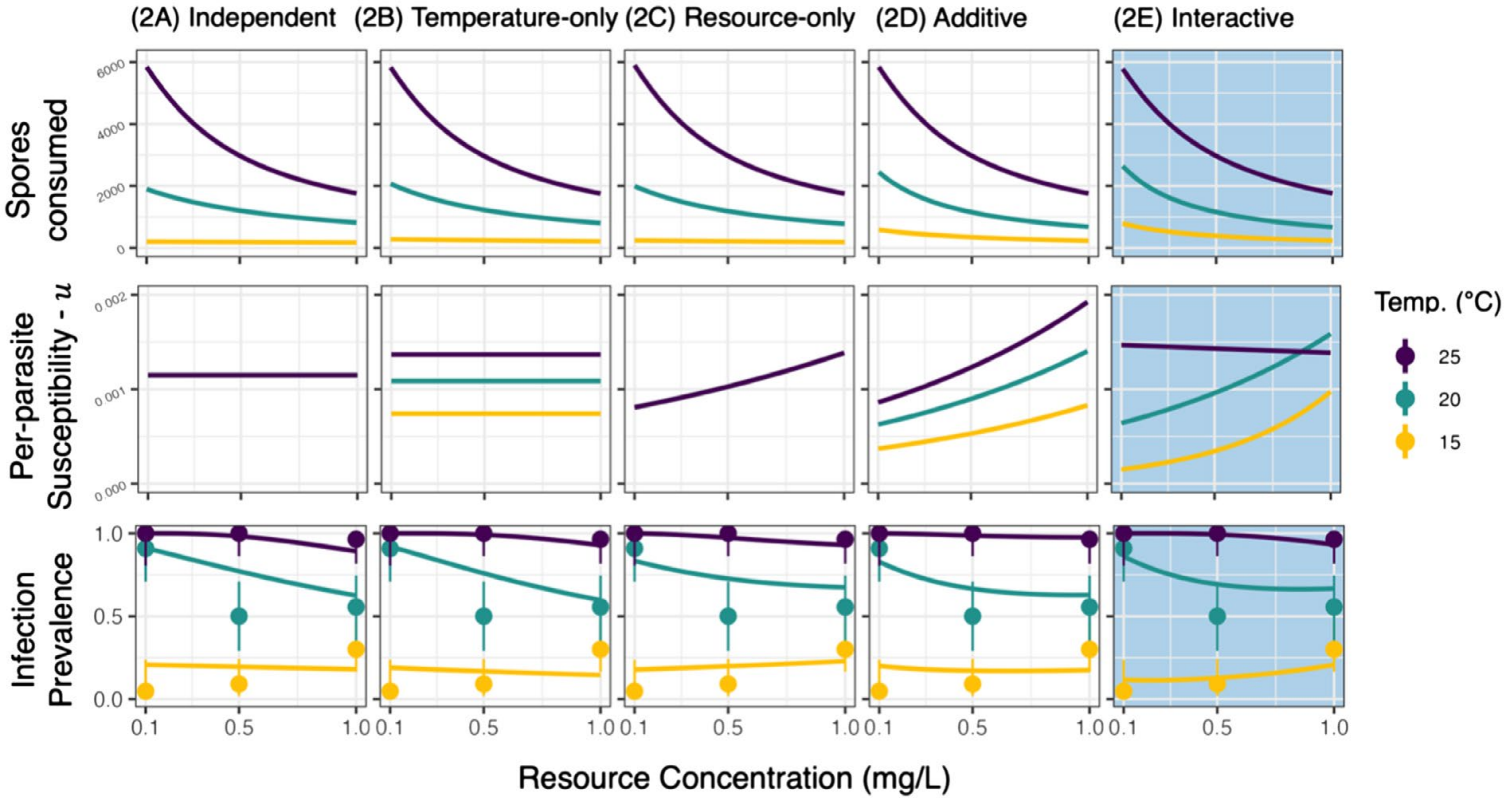
Five separate models from model competition 2 fit to the infection data. Columns show the best fit for each model: (1A) Independent; (1B) temperature‐only; (1C) resource‐only; (1D) additive; (1E) and interactive. Top row: Spores consumed in the 24‐h exposure assuming foraging rates estimated by the best fit foraging model (1E) from model competition 1. Middle row: Per‐parasite susceptibility as estimated by each model formulation. Bottom row: Predicted and observed infection prevalence from the infection assay. Error bars represent 95% confidence intervals from a binomial distribution. The best fitting model (2E) is shaded in blue.

Each of the models generated patterns of infection that somewhat resembled the experimental data by virtue of including the top model for foraging (i.e., contact rate; see Model Competition 1). However, each of these models sought to explain additional variation in the infection assay by specifying functional forms for u (Figure 3). The independent (2A) and temperature‐only models (2B) performed worst and were unable to capture the opposing trends in infection prevalence across resources at 15°C and 20°C. The resource‐only (2C) and additive models (2D) performed slightly better, and the interactive model (model 2E) clearly performed best. It was the only model able to capture the increase in infection prevalence with resources at 15°C, the reduction in prevalence with resources at 20°C, and universally high prevalence with resources at 25°C (Figure 3, bottom‐right).

**FIGURE 4:**
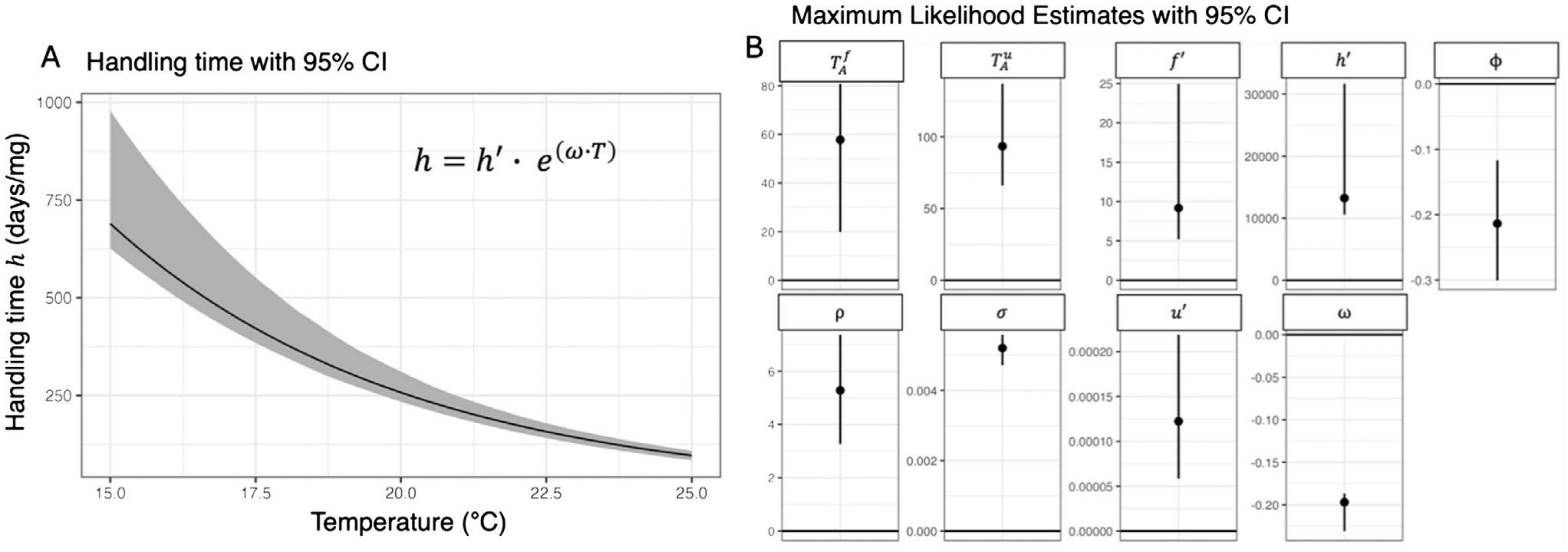
Bootstrapped confidence intervals for parameters of the winning model in Competition 2. (A) Warmer temperature reduced handing time, as specified by an exponential function in the best fitting foraging rate model (1E). Ribbons represent 95% bootstrapped confidence intervals (1005 iterations). (B) Maximum likelihood estimates with bootstrapped 95% confidence intervals for all parameters from the top performing model (model 2E), including the Arrhenius coefficient for foraging rate ($T_{A}^{f}$), the Arrhenius coefficient for per‐parasite susceptibility ($T_{A}^{u}$), size‐specific foraging rate ($f^{\prime }$), handling time at 0C ($h^{\prime }$), the linear temperature × resource interaction term for per‐parasite susceptibility (ϕ), the slope of the relationship between resources and per‐parasite susceptibility (ρ), the standard deviation of square‐root transformed fluorescence data (σ), per‐parasite susceptibility at the reference temperature and no resources ($u^{\prime }$), and the effect of temperature on handling time (ω).

**FIGURE 5:**
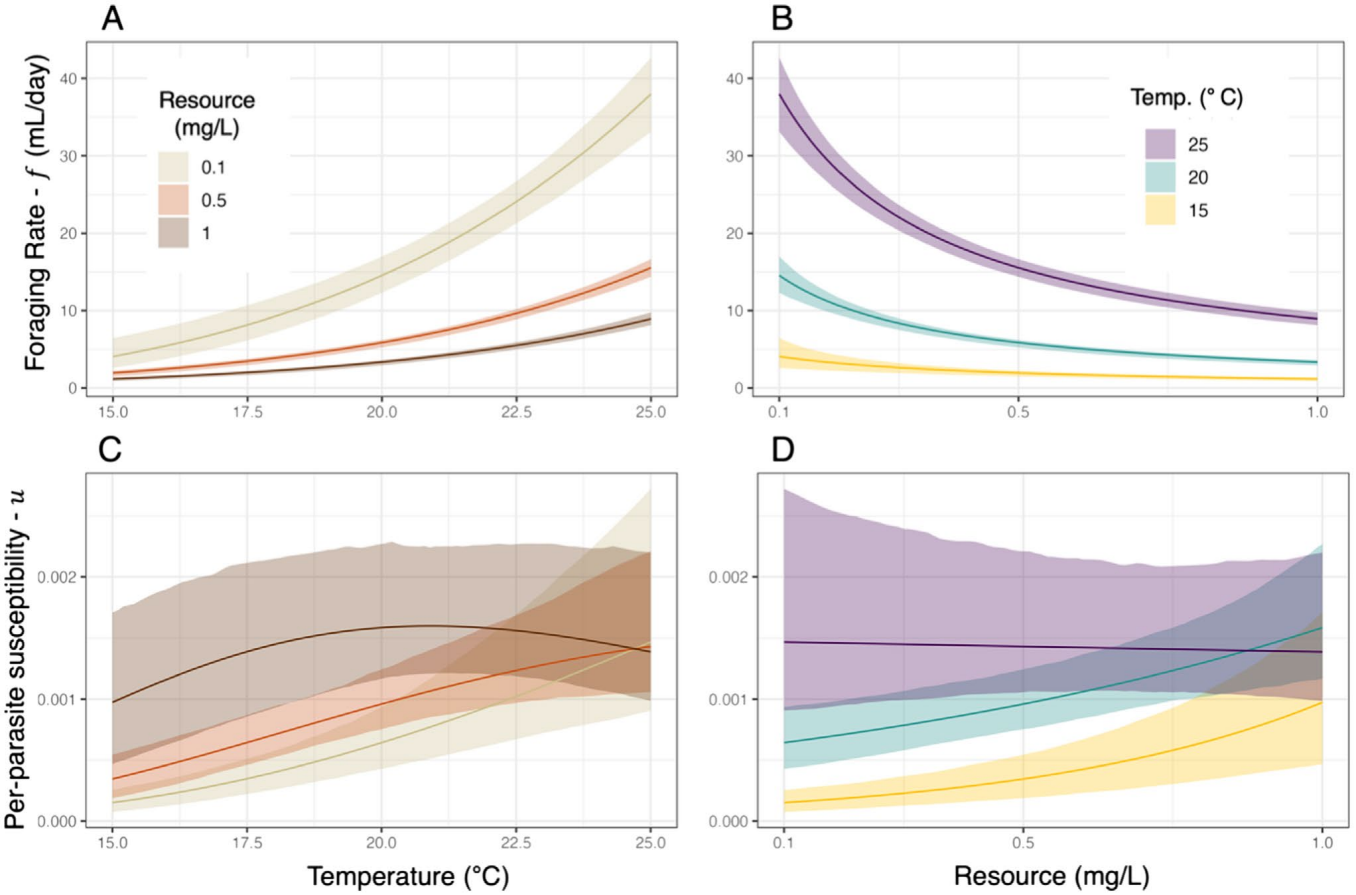
Bootstrapped confidence intervals (95%) for foraging rate and per‐parasite susceptibility from the top performing model (model 2E). Ribbons are bounded by the 2.5 and 97.5 percentile values across the *x*‐axis from simulations using data from 1005 unique stratified bootstrapped subsets of the data. Top row: (A) Foraging rate estimate for each resource condition across temperature. (B) Foraging rate estimate for each temperature condition across resources. Bottom row: (C) Per‐parasite susceptibility for each resource condition across temperature. (D) Per‐parasite susceptibility for each temperature condition across resources.

Overall, the model that incorporated both interactive effects of temperature and resources on foraging rate and interactive effects of temperature and resources on per‐parasite susceptibility best explained the data (i.e., model 2E). Furthermore, 95% confidence intervals around all parameter estimates for model 2E excluded 0 (Figure 4B). These results were qualitatively similar when per‐parasite susceptibility also varied with body size (Figure S2). Foraging rate increased with temperature and decreased with resources, and this resource effect was more gradual at warmer temperatures (Figure 5A,B). This interaction aligns with expectations of a type‐II functional response and temperature‐dependent handling time. Per‐parasite susceptibility, by contrast, did not decline with resources as we had predicted. Instead, per‐parasite susceptibility increased along a resource gradient at 15°C and 20°C and was consistently higher and less sensitive to resources at 25°C (Figure 5D). This produced resource‐dependent responses of per‐parasite susceptibility to temperature (Figure 5C), including monotonic increasing (15°C and 20°C) and unimodal relationships (25°C). Consequently, we found clear interactions between resources and temperature for both processes underlying transmission.

## 4 Discussion

We developed mechanistic models that incorporate effects of both temperature and resources on disease transmission and then tested these models against experimental data. Across disease systems, temperature can enhance contact rates between hosts and infectious propagules or individuals (Mordecai et al. 2017; Shocket et al. 2018) and affect both the susceptibility of hosts (Catalán et al. 2012) and the infectivity of parasites (Cohen et al. 2017, 2019). Resources can also affect disease transmission via changes in foraging behaviour (Becker and Hall 2014; Guindre‐Parker et al. 2022; Hall et al. 2007) and immunity (Budischak et al. 2018; Houston et al. 2007; Strandin et al. 2018). Here, we found that temperature and resources interacted to shape both contact rates and the probability of infection given contact in a model zooplankton‐fungus study system. Overall, this model fitting process illustrates how temperature and resources interact to shape transmission in this system and introduces a general mechanistic framework that could be modified to synthesise effects of resources and temperature in other disease systems.

Foraging ecology affects the transmission of a wide variety of infectious diseases, including those caused by trophically‐transmitted and vector‐borne parasites (Hartemink et al. 2015; Luong et al. 2014). For environmentally transmitted parasites, both changes in resource concentrations and temperature can independently shape disease dynamics via host foraging rate (Hall et al. 2007; Shocket et al. 2018). We synthesised these bodies of research by considering how resources and temperature jointly shape foraging and disease. We described these effects mathematically with general, mechanistic relationships: a power function to relate foraging rate and body size, a type‐II functional response to incorporate the effect of resource density, and an Arrhenius function to incorporate the effect of temperature. Importantly, we detected an interaction between temperature and resources that emerged because handling time (a key feature of type‐II foraging) decreased exponentially with temperature. In other ectotherms that exhibit type‐II functional responses, such as the ladybeetle–aphid (Sentis et al. 2013) and flatworm–paramecium (Robertson and Hammill 2021) systems, similar effects of temperature on handling time have been observed. Thus, interactions between temperature and disease could be broadly relevant for any invertebrate host or vector that encounters parasites while foraging.

Per‐parasite susceptibility (u) was also shaped by an interaction between temperature and resources. It is important to note that we modelled u as a host trait, but it can also represent per‐parasite infectivity which has been shown to increase with temperature in the same *Daphnia‐Metschnikowia* study system (Shocket et al. 2018). In other systems such as trematodes that infect snails (Studer et al. 2010) and mosquito‐borne diseases (Mordecai et al. 2019), the probability of infection increases with temperature up to a thermal optimum and then decreases. In contrast, the effects of resources on per‐parasite susceptibility that we uncovered were somewhat surprising. The interaction between temperature and resources revealed that per‐parasite susceptibility increased with resources at 15°C and 20°C but declined with resources at 25°C. This resource effect is likely related to immunity‐related host traits such as gut penetrability or hemocyte responses. Generally, higher resource availability results in greater investment in immunity, reducing host susceptibility (Strandin et al. 2018). Interestingly, prior results in the *Daphnia* system found that thicker gut epithelia were more easily penetrated by spores (Stewart Merrill et al. 2019). Although we did not measure gut thickness in our experiment, it is possible that hosts developed thicker gut epithelia with the combination of higher resources and cooler temperatures, potentially resulting in greater susceptibility of these hosts. Higher resources could also promote infection if the parasite—which steals resources from its host—benefits more (Bittner et al. 2002; Hall et al. 2009). Future studies are needed to quantify how temperature and resources combine to shape gut thickness, gut penetrability, and hemocyte responses in this system and how these factors correlate with host body size (Stewart Merrill et al. 2019; Sun et al. 2023). Similar effects of temperature and resources on host susceptibility could be relevant for other ectothermic species such as mosquitoes (Merritt et al. 1992; Mordecai et al. 2019) and snails (Civitello et al. 2020; Kalinda et al. 2017) in which transmission is known to depend on both temperature and resources separately (Huxley et al. 2021).

Ideally, resource‐dependent thermal performance curves could enable a more robust, mechanistic analysis of the interactions between temperature and resources on both foraging rate and per‐parasite susceptibility. Thermal performance curves have been established as an invaluable tool for predicting range expansions and contractions of disease vectors (Mordecai et al. 2017; Ryan et al. 2019) and assessing differential effects of temperature on hosts and their parasites (Cohen et al. 2017; Gehman et al. 2018). However, these analyses generally do not account for interactions between temperature and resources. If changes in temperature due to climate change are coupled with changes in resource availability, then the incorporation of resource effects on thermal performance may be necessary for accurately predicting effects of climate change. Our 3 × 3 factorial experimental design did not allow for the parameterisation of resource‐dependent thermal performance curves. We intentionally selected a set of temperatures and resource concentrations that these hosts experience in nature and were likely to survive. However, a larger experiment might include treatments that spanned critical thermal minima and maxima (i.e., causing host/parasite mortality), or by analogy, a critical resource minimum (i.e., causing starvation). A broader design such as this would enable us to ask whether resource concentrations shift the thermal optima of traits such as host foraging rate, handling time, and per‐parasite susceptibility. With this approach we could ask, for example, does warmer temperature always reduce host handling time, or does it begin to increase again before the critical thermal maximum? This approach might also bring mechanistic clarity to the surprising interaction we detected for per‐parasite susceptibility. Specifically, our results are consistent with a resource‐dependent thermal performance curve for per‐parasite susceptibility, with higher resource availability pushing the thermal optimum of *u* down to lower temperatures (e.g., near 20°C).

Our study leaves room for additional future expansions. First, a similar experiment that also manipulated parasite dose could explore how host immunity varies with different ratios of resources and spores consumed. In this system, foraging and exposure are correlated, but spore gradients could break this correlation and serve as a better model for other study systems in which these processes are less tightly coupled. Second, performing this experiment across multiple host genotypes would test whether these patterns persist across multiple genotypes (Auld et al. 2012; Duffy and Sivars‐Becker 2007). If host genotypes differ in these responses, then differences in temperature and/or food availability could drive different trajectories of host evolution. Such evolutionary responses seem likely in nature, where both temperature and resources change seasonally. Third, the effects of temperature fluctuations are also important for understanding transmission and cannot be detected in experiments conducted under constant temperature conditions (Ferguson and Sinclair 2020; Paaijmans et al. 2013; Vasseur et al. 2014). We could incorporate temperature variation to observe how realistic variations in temperature might alter these results since *Daphnia* and many hosts experience variable environments (Dallas and Drake 2016). These future expansions can further corroborate our results and add to our theoretical understanding of temperature and resource interactions in host–parasite systems.

Interactions between temperature and resources are important for understanding disease transmission in a variety of systems. Anthropogenic effects such as climate change, eutrophication, and urbanisation are likely to alter both temperature and resources simultaneously, enhancing the need to understand the interaction between these factors (Altizer et al. 2013; Becker et al. 2015; Bradley and Altizer 2007). Other systems with ectothermic hosts or vectors where foraging and transmission are linked may exhibit similar patterns to the *Daphnia–Metschnikowia* system, and these models can be adapted to other forms of transmission as well. Altogether, these models and empirical examples can enhance our fundamental understanding of environmental effects on disease transmission and provide a framework for incorporating the effects of multiple environmental variables.

## Author Contributions

Daniel C. Suh, Katie Schroeder and Alexander T. Strauss designed the study. Daniel C. Suh and Katie Schroeder collected the data. Daniel C. Suh performed the analysis, with guidance from Alexander T. Strauss. Daniel C. Suh wrote the first draft of the manuscript, and all authors contributed to the revisions.

## Peer Review

The peer review history for this article is available at https://www.webofscience.com/api/gateway/wos/peer‐review/10.1111/ele.70151.

## Supporting information


Data S1.

## Data Availability

All code and data are available at https://osf.io/j3bvu/.
