# Supplementary material for: Temperature and Resources Interact to Affect Transmission via Host Foraging Rate and Susceptibility
Source: Ecol Lett. 2025 Jun 24;28(6):e70151. doi: 10.1111/ele.70151 (PMC12186588; doi:10.1111/ele.70151)
Supplement: Supplementary file 1 — Data S1. [file ELE-28-0-s001.docx]

**Supplementary Materials**

**Title:** Temperature and resources interact to affect transmission via host foraging rate and susceptibility

**Authors:** Daniel C. Suh^1,2,3^, Katie Schroeder^1^, Alexander T. Strauss^1,2^

^1^Odum School of Ecology, University of Georgia, Georgia, United States of America

^2^Center for the Ecology of Infectious Diseases University of Georgia, Georgia, United States of America

^3^Current address: Department of Biological Sciences, Virginia Tech, Blacksburg, Virginia, United States of America

In this appendix, we present supplementary materials regarding:

(A) experimental details (Methods S1; Table S1)

(B) body length measurements (Methods S2; Figure S1)

(C) model competitions 1 and 2 (Methods S3-4; Tables S2-3)

(D) traditional statistics (Table S4)

(E) size-dependent per-parasite susceptibility model (Methods S5; Figures S2-3)

1. ***Experimental Details***

**Supplementary Methods S1: Estimating resource concentration from fluorescence**

For stock solutions of the resource, a standard curve was generated to relate absorbance of the resource in solution to dry weight of algae after filtering onto filter paper (1µm Pall A/E). Algae was harvested in batch and then diluted to a standard concentration using the standard curve, resulting in concentrations in units of mg dry mass algae per liter of water (mg/L). In-vivo fluorescence of both grazed and ungrazed tubes in the foraging assay was measured using a Turner Trilogy fluorometer in relative fluorescence units (RFU’s). Ungrazed control tubes represented the initial resource concentration in the treatment (grazed) tubes. Actual resource concentrations in the control tubes varied and differed slightly from the nominal treatment levels (0.1, 0.5, or 1.0 mg/L), so we used linear regressions to convert RFU readings of control tubes back to units of actual mg/L to more precisely identify actual resource concentrations used in each treatment of the experiment. We plotted intended resource concentration (mg/L) against fluorescence readings in control tubes (RFUs) and conducted linear regression across treatments. Actual resource concentrations (mg/L) were estimated as the fluorescence readings (RFUs) divided by the slope of this regression.

**Table S1: Sample sizes and infection outcomes of the infection assay (n = 237)**

| Temperature (°C) | Resource (mg L^-1^) | n | # infected |
| --- | --- | --- | --- |
| 15 | 0.1 | 21 | 1 |
| 15 | 0.5 | 33 | 3 |
| 15 | 1.0 | 40 | 12 |
| 20 | 0.1 | 22 | 20 |
| 20 | 0.5 | 24 | 12 |
| 20 | 1.0 | 27 | 15 |
| 25 | 0.1 | 17 | 17 |
| 25 | 0.5 | 25 | 25 |
| 25 | 1.0 | 28 | 27 |

1. ***Body Length measurements***

**Supplementary Methods S2: Body length measurement and interpolation**

In the foraging assay, body length was measured directly whenever possible, resulting in a range of 22-28 individuals per treatment that could be measured. Length was estimated using a dissecting microscope and eyepiece micrometer. If individuals were not able to be measured due to handling errors, then body length was interpolated as the mean length from that treatment. This applied to 41 out of a total of 270 individuals. Interpolated body lengths were used in the dynamical models but were omitted from the traditional statistics.

For the infection assay, body length was estimated for each treatment by averaging the body length of 7-10 extra individuals that were not included in the experiment. The 25°C 0.5mg/L treatment did not have enough extra individuals to be used for length measurements (i.e., all individuals were used in the experiment), so length was estimated as 1.16mm, the average between lengths in the 25°C 0.1mg/L and 25°C 1.0mg/L treatments. In order to plot model predictions over continuous gradients of temperature and resources (e.g., Figs. 2 & 3), we needed estimates of body size under these other conditions. We interpolated these body sizes between treatment conditions using multiple linear regression, with additive effects of temperature and resources. We interpolated body size using the coefficients from the model. This model fit well when applied to body sizes from the foraging assay (R^2^ of 0.8832) and body sizes in the infection assay (R^2^ of 0.7058), providing robust estimates of body size under any combination of temperature and resources.

**
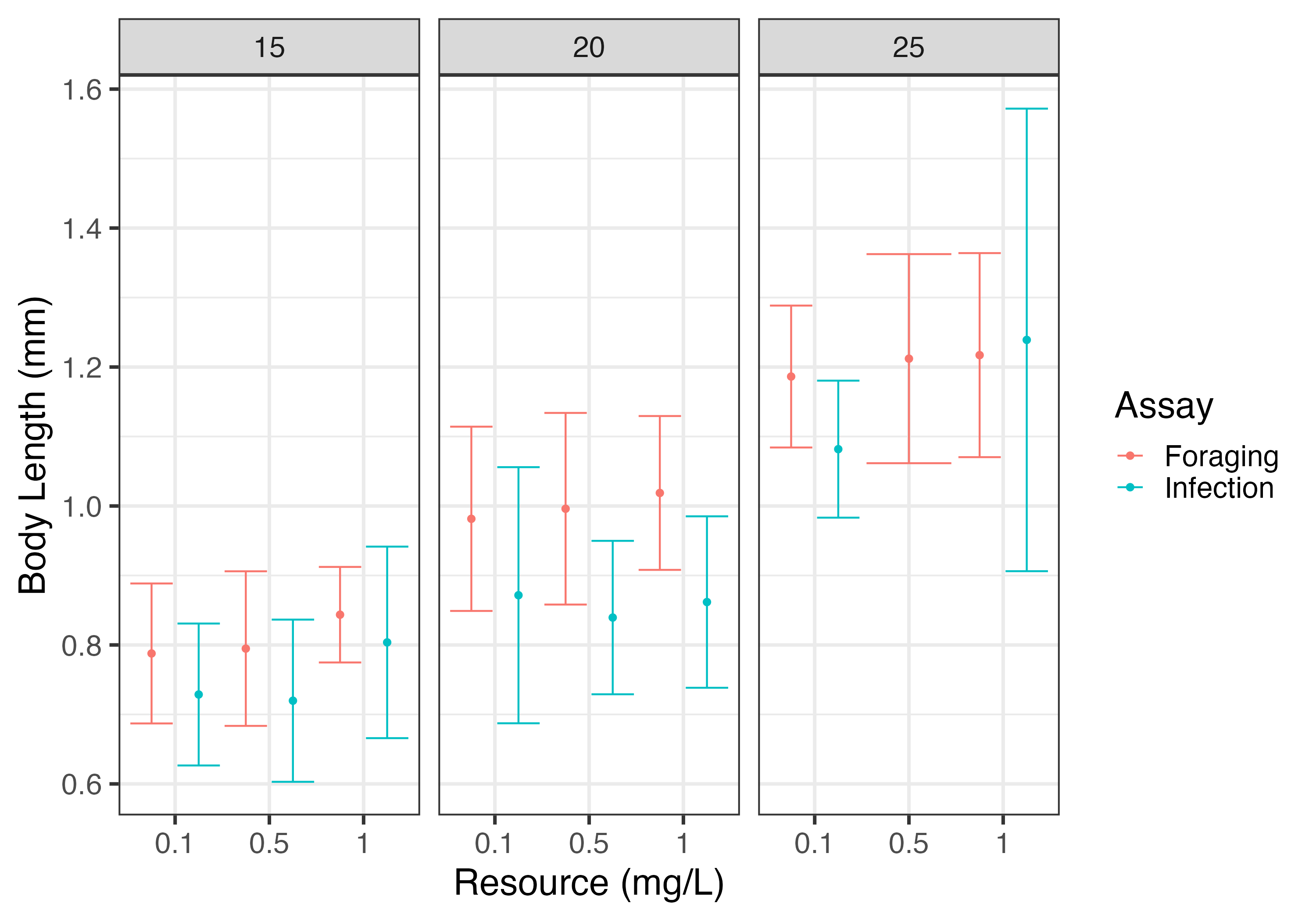
**

**Figure S1: Mean length measurements from foraging and infection assay.** Error bars represent +/- two standard deviations of the mean. Facets represent each temperature treatment. Sample sizes for the foraging assay ranged from 22-28 individuals. Sample sizes for the infection assay ranged from 7-10 individuals. Body length data from the foraging assay were used for model competition 1, resulting in size-specific foraging parameters. In model competition 2, we again used body length data from the foraging assay to maximize likelihood of observing the results of the foraging assay, again yielding size-specific foraging parameters. However, we used body length data from the infection assay, along with the same size-specific foraging parameters, to maximize likelihood of observing the outcomes of the infection assay. Thus, although body size was slightly different between the two assays, we accounted for this difference in our model fitting process.

1. ***Model competition 1 and 2***

**Supplementary Methods S3: Model formulations for foraging rate**

$\frac{dR}{dt}= -fRN$ (1)

In each model, we replace $f$ from equation 1 with a function of $f^{'}$ and other parameters. We use the notation of $f^{'}$ throughout our models, but the interpretation of this parameter differs slightly between each model. Across the models, $f^{'}$ is the size-specific maximum foraging rate at certain treatment conditions (temperature, resource, or both).

(**Model 1A – size-only** $\boldsymbol{f}$) The simplest formulation described foraging rate across all treatments using a single parameter $f^{'}$ and a power function for host surface area (body length [*L*] squared):

$f= f^{'}L^{2}$ (1A)

This model assumes that the variation in foraging rate is solely due to the variation in body size that resulted from the growth at treatment conditions prior to the start of the assay (up to day 5). Each of the following models assume the same power function for body length.

(**Model 1B – temperature-only** $\boldsymbol{f}$) Temperature-dependence was modeled using the Arrhenius function:

$f= f^{'}L^{2}e^{T_{A}^{f}\left( 1/T_{R}-1/T \right)}$ (1B)

The Arrhenius function modifies foraging rate as an exponential function of temperature that scales foraging rate at a reference temperature ($T_{R}$) according to the treatment temperature $(T$) via a constant slope $T_{A}^{f}$. Functionally, this form of the Arrhenius function captures the exponentially increasing portion of a thermal reaction norm. We chose this function because of its simplicity and ability to model effects of our chosen temperature range, which covers a majority of the thermal range over which *Daphnia* survive (Hall et al., 2006). In this model, the interpretation of $f^{'}$ is the size-specific foraging rate at the reference temperature.

(**Model 1C – resource-only** $\boldsymbol{f}$) Resource-dependence was modeled as a type-II functional response (Sarnelle & Wilson, 2008):

$f=\frac{f^{'}L^{2}}{1+f^{'}L^{2}hR}$ (1C)

Here, foraging rates are modified by the incorporation of a handling time $h$ that describes the time required to “handle” (i.e., capture and process) each unit of resource. Handling time causes foraging rate to slow and then asymptote at higher resource concentrations. We chose to use the handling time formulation of the type-II functional response rather than the half-saturation formulation because we preferred the mechanistic interpretation of a handling time. This form alters the interpretation of $f^{'}$, which here represents the maximum size-specific foraging rate when resource concentration is 0 (or handling time is 0).

(**Model 1D – additive** $\boldsymbol{f}$) The additive model combines models 1B and 1C by modifying foraging rate according to the Arrhenius function (in the numerator) while maintaining a type-II functional form (in the denominator):

$f=\frac{f^{'}{L^{2}e}^{T_{A}^{f}\left( 1/T_{R}-1/T \right)}}{1+f^{'}L^{2}e^{T_{A}^{f}\left( 1/T_{R}-1/T \right)}hR}$ (1D)

Using this formulation, the effects of both temperature and resources are included but independent of each other. The temperature effect does not change along the resource gradient and the resource effect does not change along the temperature gradient. In this instance, $f^{'}$ represents the maximum size-specific foraging rate at the reference temperature when resources are absent.

(**Model 1E – interactive** $\boldsymbol{f}$) Finally, we built upon the additive model by including an additional term that allows for an interaction between temperature and resource, with handling time itself as an exponential function of temperature:

$h=h^{'}e^{\omega T}$ (1E)

In this model, foraging rates vary with both temperature and resources, and furthermore temperature modifies the functional response by altering handling time. This interactive model is the most complex foraging rate model and includes five total parameters: body size *L*, foraging rate $f^{'}$, the Arrhenius constant $A_{T}$, temperature-dependent handling time $h'$, and an interaction term $\omega$ that describes how temperature affects handling time. Handling time increases with temperature if $\omega>0$ and decreases with temperature if $\omega<0$ and is equivalent to handling time in model 1D when $\omega=0$.

**Supplementary Methods S4: Model formulations for per-parasite susceptibility**

$\frac{dR}{dt}= -fR\left( S+I \right)$ (2)

$\frac{dS}{dt}= -ufZS$

$\frac{dI}{dt}= ufZS$

$\frac{dZ}{dt}= -fZ\left( S+I \right)$

We replaced $u$ with $u^{'}$ and other terms to incorporate the effects of temperature and/or resources. Similarly to $f^{'}$, the interpretation of $u^{'}$ depends on the model formulation and is the per-parasite susceptibility at certain treatment conditions (temperature, resources, or both).

(**Model 2A – independent** $\boldsymbol{u}$) The simplest model assumes that $u$ is independent and does not change across treatments:

$u = u^{'}$ (2A)

Therefore, differences in infection outcomes are driven exclusively by effects of temperature and resources via host foraging (i.e., contact rates).

(**Model 2B – temperature-only** $\boldsymbol{u}$**)** Temperature dependence for per-parasite susceptibility was also modeled using the Arrhenius function and incorporates the effect of temperature on $u$ according to an infection-specific constant $T_{A}^{u}$:

$u = u^{'}e^{T_{A}^{u}\left( 1/T_{R}-1/T \right)}$ (2B)

Under this formulation, the effect of temperature can be positive or negative depending on the sign of $T_{A}^{u}$ but cannot generate a non-monotonic relationship with per-parasite susceptibility. When $T_{A}^{u}$ is positive, per-parasite susceptibility increases with temperature (e.g., parasite infectivity benefits from warmer temperature) and vice versa when $T_{A}^{u}$ is negative (e.g., host immunity benefits from warmer temperature). The parameter $u^{'}$ in this model can be interpreted as the per-parasite susceptibility at the reference temperature.

(**Model 2C – resource-only** $\boldsymbol{u}$) Resource dependence was modeled with per-parasite susceptibility as an exponential function of the treatment’s resource conditions $\left[ R_{trt} \right]$:

$u=u^{'}e^{\rho\left[ R_{trt} \right]}$ (2C)

We chose to use experimental resource conditions (a constant) rather than the state variable $R$ (which decreased dynamically in the models) because we assumed that any effects of resources on per-parasite susceptibility resulted from cumulative differences in food consumption over the first five days of the host’s life (e.g., altered immune function). The addition of $\rho$ allows per-parasite susceptibility to increase or decrease with resources. A positive $\rho$ would indicate an increase in susceptibility across resources, a pattern consistent with previous experiments in this system (Stewart Merrill et al., 2019), potentially due to altered physiology and penetrability of the host gut. A negative $\rho$ would indicate declining per-parasite susceptibility with resources and might indicate stronger host immune function due to enhanced resource consumption. In this model, $u^{'}$ is the per-parasite susceptibility when resources are absent.

(**Model 2D – additive** $\boldsymbol{u}$) The additive version of the transmission model is a combination of models G and H:

$u=u^{'}e^{T_{A}^{u}\left( 1/T_{R}-1/T \right) + \rho\left[ R_{trt} \right]}$ (2D)

This model includes effects of both temperature and resources on per-parasite susceptibility but does not include interactive effects. Here, $u^{'}$ is the per-parasite susceptibility at the reference temperature when resources are absent.

(**Model 2E – interactive** $\boldsymbol{u}$) Finally, we developed an interactive model that included an additional parameter to permit interactive effects of resources and temperature on per-parasite susceptibility:

$u=u^{'}e^{T_{A}^{u}\left( 1/T_{R}-1/T \right) + \rho\left[ R_{trt} \right] + \phi\left[ R_{trt} \right]T}$ (2E)

The inclusion of $\phi$ allows for the resource effect on per-parasite susceptibility to vary between temperature treatments and vice versa. This model is the most complex of all the competing models.

**Table S2: State variables and parameters**

| **Term** | **Units** | **Models** | | **Definition** |
| --- | --- | --- | --- | --- |
| State variables |  | Comp. 1 | Comp. 2 |  |
| $S$ | Hosts L^-1^ | all | | Susceptible Hosts |
| $I$ | Hosts L^-1^ | all | | Infected Hosts |
| $R$ | mg L^-1^ | all | | Algal concentration |
| $Z$ | Spores L^-1^ | all | | Fungal spores |
| Parameters |  | Comp. 1 | Comp. 2 |  |
| $f$ | L day^-1^ | all | | Foraging rate |
| $L$ | mm | all | | Host body length |
| $f'$ | L day^-1^ mm^-2^ | all* | | Size-specific foraging rate* |
| $T$ | °Celsius | 1B, 1D-E | 2A-E | Treatment temperature |
| $T_{R}$ | °Celsius | 1B, 1D-E | 2A-E | Reference temperature (15°C) |
| $T_{A}^{f}$ | °Celsius | 1B, 1D-E | 2A-E | Arrhenius Temperature for $f$ |
| $h$ | Days mg^-1^ | 1C-E | 2A-E | Handling time |
| $h'$ | Days mg^-1^ | 1E | 2A-E | Handling time at 0°C |
| $\omega$ | °Celsius^-1^ | 1E | 2A-E | Temperature effect on $h'$ |
| $u$ | Infections spore^-1^ | - | 2A-E | Per-parasite susceptibility |
| $u'$ | Infections spore^-1^ | - | 2A-E* | Adjusted per-parasite susceptibility* |
| $T_{A}^{u}$ | °Celsius | - | 2B, 2D-E | Arrhenius temperature for $u$ |
| ${[R}_{trt}$] | mg L^-1^ | - | 2C-E | Treatment Resource level |
| $\rho$ | L mg^-1^ | - | 2C-E | Resource effect on $u$ |
| $\theta$ | L mg^-1^ °Celsius^-1^ | - | 2E | Interactive effect on $u$ |
| $\sigma$ | NA | all | | Standard deviation |

*Interpretation and units of $f^{'}$ and $u^{'}$ depends on the model formulation, since adding a slope changes the meaning of the intercept. Units shown are for models 1A and 2A, respectively.

**Table S3: Parameter Estimates and Bootstrapped confidence intervals from the top performing model (n=1005)**

| Bootstrapped 95% Confidence Intervals | | | |
| --- | --- | --- | --- |
| Parameter | Estimate | 2.5% | 97.5% |
| $f'$ | 9.168 | 5.2163 | 24.9405 |
| $u'$ | 12.2426E^-5^ | 5.8436E^-5^ | 21.8968E^-5^ |
| $T_{A}^{f}$ | 57.7777 | 19.9025 | 80.7915 |
| $T_{A}^{u}$ | 93.3982 | 65.9484 | 136.9036 |
| $h'$ | 13224.9606 | 10577.7898 | 31639.3035 |
| $\omega$ | -0.1969 | -0.2308 | -0.1868 |
| $\rho$ | 5.2786 | 3.2678 | 7.3671 |
| $\theta$ | -0.2137 | -0.3003 | -0.1169 |
| $\sigma$ | 0.0052 | 0.0047 | 0.0056 |

1. ***Traditional Statistics***

**Table S4: Results from traditional statistical analysis**

**Length ~ resource + temperature (df = 226)**

| Predictor | Estimate | Std. Error | t value | P-value |
| --- | --- | --- | --- | --- |
| Intercept | 0.188 | 0.020 | 9.310 | <0.001 |
| Resource | 0.046 | 0.011 | 4.332 | <0.001 |
| Temperature | 0.040 | 0.001 | 41.228 | <0.001 |

**Resources consumed ~ resource * temperature (df = 267)**

| Predictor | Estimate | Std. Error | t value | P-value |
| --- | --- | --- | --- | --- |
| Intercept | -0.050 | 0.026 | -1.913 | 0.057 |
| Resource | -0.399 | 0.040 | -9.862 | <0.001 |
| Temperature | 0.004 | 0.001 | 3.086 | 0.002 |
| Resource:  Temperature | 0.024 | 0.002 | 12.325 | <0.001 |

**Resources consumed ~ resource**

| Temp | Predictor | Estimate | Std. Error | t value | P-value | df |
| --- | --- | --- | --- | --- | --- | --- |
| 15 | Intercept | 0.007 | 0.009 | 0.773 | 0.442 | 88 |
| 15 | Resource | -0.026 | 0.015 | -1.807 | 0.074 |  |
|  |  |  |  |  |  |  |
| 20 | Intercept | 0.033 | 0.008 | 4.283 | <0.001 | 88 |
| 20 | Resource | 0.077 | 0.012 | 6.457 | <0.001 |  |
|  |  |  |  |  |  |  |
| 25 | Intercept | 0.047 | 0.010 | 4.711 | <0.001 | 88 |
| 25 | Resource | 0.218 | 0.015 | 14.183 | <0.001 |  |

**Probability of Infection ~ resource * temperature (df = 233)**

| Predictor | Estimate | Std. Error | z value | P-value |
| --- | --- | --- | --- | --- |
| Intercept | -18.338 | 3.625 | -5.059 | <0.001 |
| Resource | 0.989 | 0.190 | 5.213 | <0.001 |
| Temperature | 12.357 | 4.076 | 3.032 | 0.002 |
| Resource:  Temperature | -0.665 | 0.213 | -3.115 | 0.002 |

**Probability of Infection ~ resource**

| Temp | Predictor | Estimate | Std. Error | z value | P-value | df |
| --- | --- | --- | --- | --- | --- | --- |
| 15 | Intercept | -3.490 | 0.876 | -3.984 | <0.001 | 92 |
| 15 | Resource | 2.623 | 1.011 | 2.595 | 0.009 |  |
|  |  |  |  |  |  |  |
| 20 | Intercept | 1.553 | 0.511 | 3.038 | 0.002 | 71 |
| 20 | Resource | -1.613 | 0.708 | -2.280 | 0.023 |  |
|  |  |  |  |  |  |  |
| 25 | Intercept | 39.906 | 11,899 | 0.003 | 0.997 | 68 |
| 25 | Resource | -36.610 | 11,899 | -0.003 | 0.998 |  |

1. ***Size-dependent infection model***

**Supplementary Methods S5: size-dependent interactive** $\boldsymbol{u}$ **model**

We did not originally plan to test a size-specific model for per-parasite susceptibility, in part because we did not have a clear mechanistic hypothesis for why this relationship should arise. Previous research in this study system showed that parasite spores are better able to penetrate the guts of hosts with thicker anterior gut epithelium, but also that a smaller fraction of the attacking spores are able to penetrate the gut of larger hosts (Stewart Merrill et al. 2019). We are unfortunately limited in our ability to test models about size-specific per-parasite susceptibility, in part because we did not measure body size of individual hosts in the infection assay (although we do have mean body size of extra hosts measured in each treatment). This limitation creates a potentially problematic issue of perfectly conflated treatment conditions and body size. When we used these identical size estimates for all individuals within a treatment, we were computationally unable to fit a model that included an additional parameter for size-specific per-parasite susceptibility. Instead, we fixed the foraging-related parameters from our best fit model (2E) and then used the infection data to fit a new infection model **(Model 2F – size-dependent** $\boldsymbol{u}$**)** that included a parameter $m$ to describe the effects of body size on per-parasite susceptibility:

$u=u^{'}e^{T_{A}^{u}\left( 1/T_{R}-1/T \right) + \rho\left[ R_{trt} \right] + \phi\left[ R_{trt} \right]T + mL}$ (2F)

This model is identical to Model 2E (with effects of temperature, resources, and their interaction) but also includes a new parameter $m$ that allows $u$ to increase or decrease with host body size $L$. A positive $m$ would indicate that per-parasite susceptibility increases with body size, while a negative $m$ would indicate that per-parasite susceptibility decreases with body size. We simulated values for spores consumed, per-parasite susceptibility, and infection prevalence across temperature and resource conditions in the same manner as the main analysis. We also simulated these values across a range of body lengths for each of our treatment conditions to observe how these responses varied with body length in the size-dependent model (Supplementary Figure S2). Finally, we bootstrapped confidence intervals (n = 100) around these parameter estimates (Supplementary Figure S3).

Bootstraps of the new model revealed that the parameter $m$ was consistently positive, indicating that per-parasite susceptibility increased with host body size. All other foraging and infection parameters were qualitatively similar to the parameters estimated by the top performing model from the model competition (2E). In other words, the positive effects were still positive, and the negative effects were still negative. Given the caveats described above (lack of individual measurements of body size and computational challenges), we view the results of this size-specific per-parasite susceptibility model as a complementary analysis to the model competitions presented in the main text. The results of this last model highlight the need for future research to mechanistically uncover how body size affects per-parasite susceptibility, and how these effects might vary with temperature or resource conditions.

**
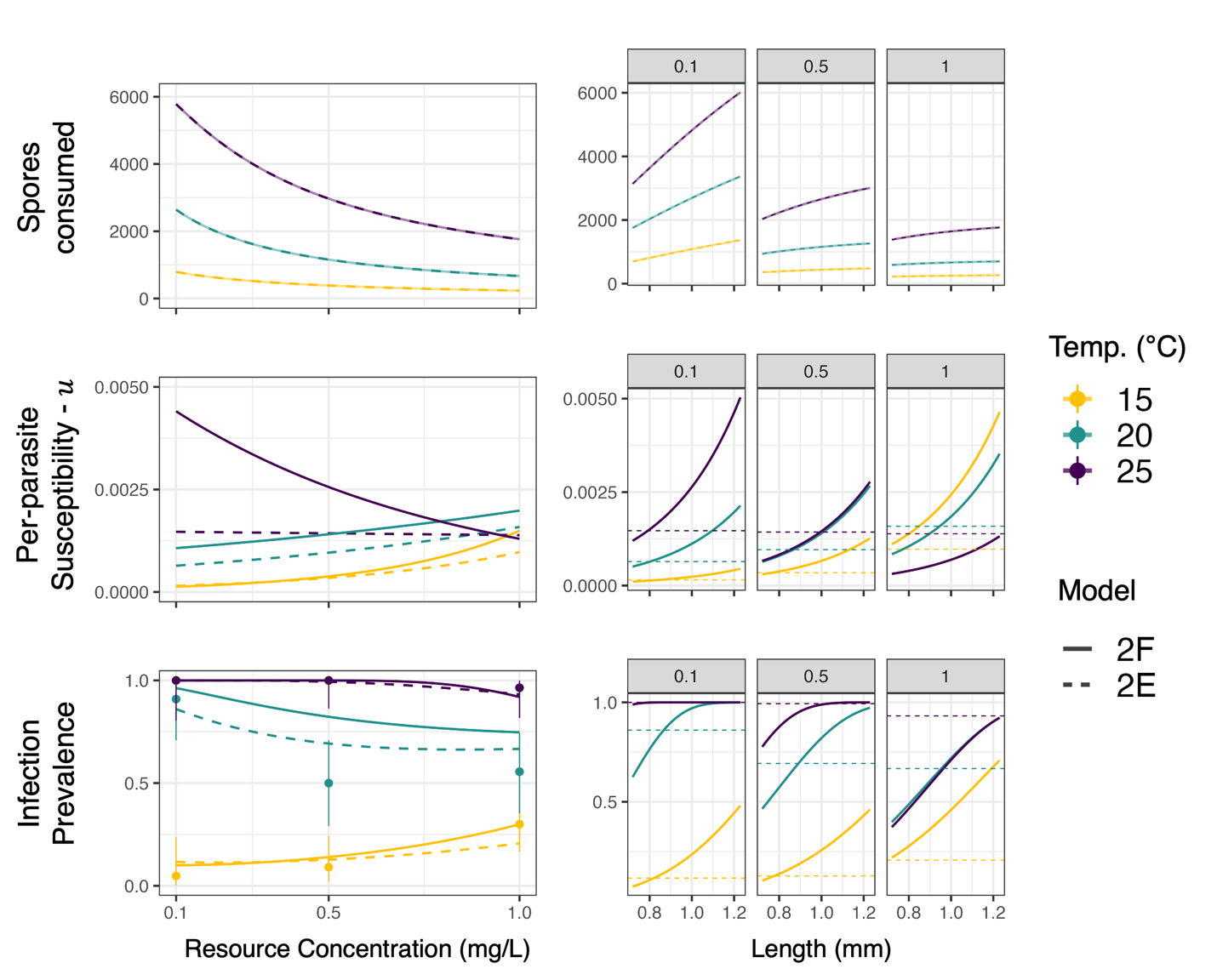
**

**Figure S2: Comparison between interactive (2E; dashed lines) and size-dependent interactive model (2F; solid lines).** Left-side panels show predicted spores consumed (top), per-parasite susceptibility (middle), and infection prevalence (bottom) for each model. Right-side panels show the same variables but visualizes their response to host body length. Spores consumed is equivalent between each model because they use the same foraging rate equation and parameter estimates. Per-parasite susceptibility and, consequently, infection prevalence change with host body length in the size-dependent model but not in the interactive model.

**
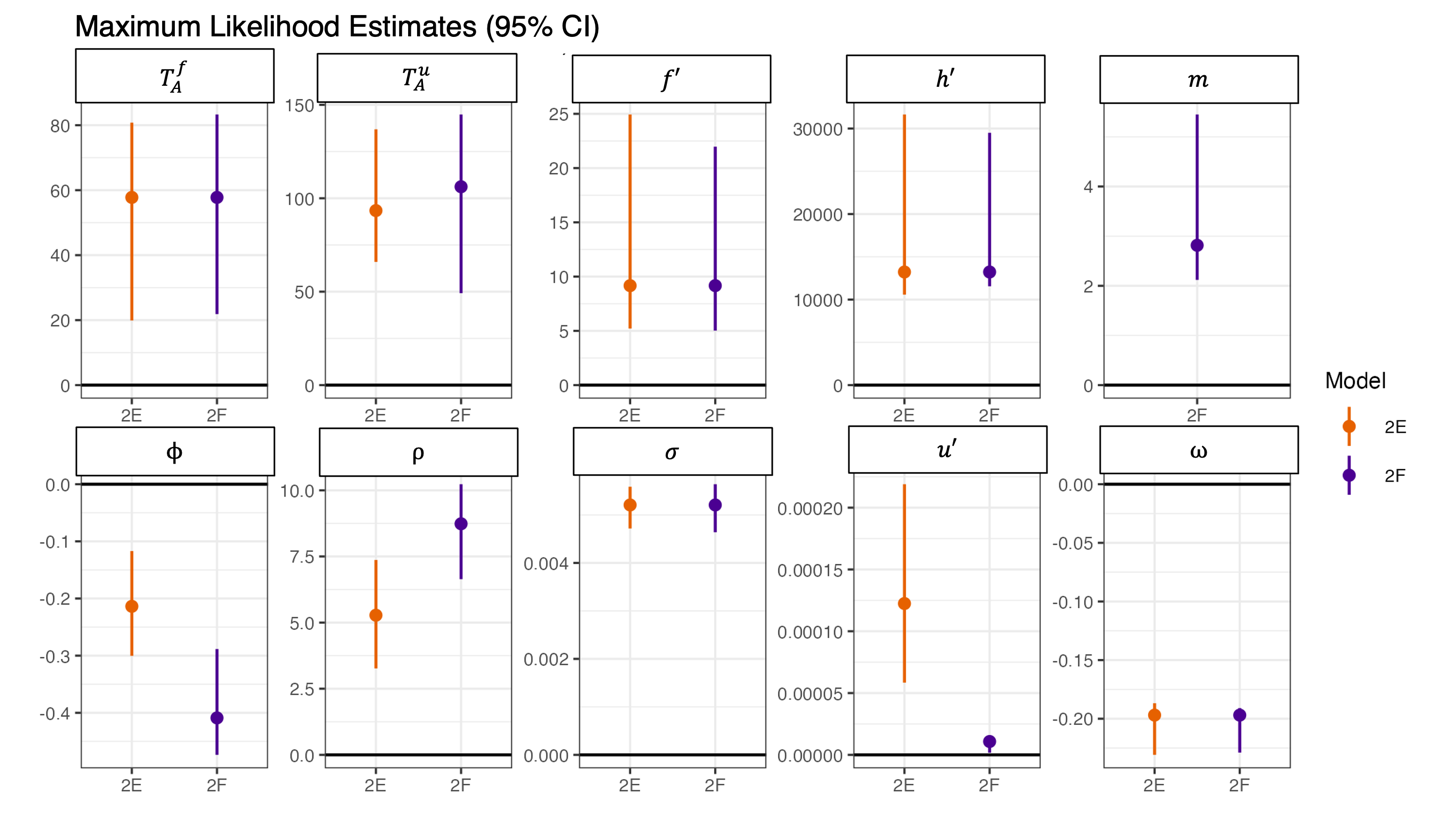
**

**Figure S3: Bootstrapped confidence intervals around parameter estimates for interactive** $\boldsymbol{u}$ **(2E) and size-dependent interactive** $\boldsymbol{u}$ **(2F) models**. Model 2F includes an additional parameter $m$ that scales per-parasite susceptibility $u$ by body length ($L$). This parameter is consistently positive, indicating that per-parasite susceptibility increases with size. Note that the difference in estimates of $u'$ between models arises because these parameters have different meanings; in the model 2F, $u'$ should be interpreted as per-parasite susceptibility of an infinitely small host. All other parameters are qualitatively similar between models 2E and 2F.
